# Respiratory virus of severe pneumonia in South Korea: Prevalence and clinical implications

**DOI:** 10.1371/journal.pone.0198902

**Published:** 2018-06-18

**Authors:** Hyung-Jun Kim, Sun Mi Choi, Jinwoo Lee, Young Sik Park, Chang-Hoon Lee, Jae-Joon Yim, Chul-Gyu Yoo, Young Whan Kim, Sung Koo Han, Sang-Min Lee

**Affiliations:** Department of Internal Medicine, Seoul National University College of Medicine, Seoul, Republic of Korea; University of North Carolina at Chapel Hill, UNITED STATES

## Abstract

**Background:**

Severe viral pneumonia is associated with a high mortality rate. However, due to the vulnerability of critically ill patients, invasive diagnostic methods should be performed with caution in the intensive care unit (ICU). It would be helpful if the prevalence, risk factors, and clinical impact of virus detection are elucidated.

**Methods:**

We evaluated patients with severe pneumonia between January 1^st^ 2008 and December 31^st^ 2015. Reverse transcription-polymerase chain reaction (RT-PCR) analysis was performed for 8 respiratory viruses when viral pathogen could not be excluded as the origin of severe pneumonia. The baseline characteristics, laboratory results, microbiological findings, and clinical outcomes of the patients were analyzed.

**Results:**

Of the 2,347 patients admitted to the medical ICU, 515 underwent RT-PCR for respiratory viruses, 69 of whom had positive results. The detection rate was higher during the winter, with a community onset, in patients with history of recent chemotherapy, and low platelet count. Additional bronchoscopic sampling along with upper respiratory specimen increased the yield of viral detection. Respiratory syncytial virus was the most common pathogen detected, while influenza A was the most common virus with bacterial coinfection. Respiratory virus detection led to changes in clinical management in one-third of the patients.

**Conclusions:**

The detection of viral pathogens in patients with severe pneumonia is not rare, and can be more common in certain group of patients. Invasive sampling for RT-PCR can be helpful, and such detection can lead to positive changes in clinical management.

## Introduction

Respiratory viruses are common pathogens in adults hospitalized with pneumonia and are more frequently detected than bacterial pathogens in certain groups of patients [1]. Influenza virus is the most well-known respiratory viral pathogen, but others, including respiratory syncytial virus (RSV) and parainfluenza virus, are also common [2, 3]. Such viral pathogens are not easily distinguishable based on clinical findings alone [4]. Therefore, it is suggested that reverse transcription-polymerase chain reaction (RT-PCR) assays should be performed in patients when a viral pathogen is suspected [5]. Such testing for respiratory viruses can decrease the inappropriate use of antibiotics and other medical resources [6].

Viral pneumonia is also a major cause of patient deterioration in the intensive care unit (ICU) [7]. Respiratory viruses are well-known for their high prevalence in patients with community acquired pneumonia (CAP) who exhibit milder clinical presentations [8]. Furthermore, their role as nosocomial pathogens in the more severely ill group of patients is being highlighted [9]. However, only limited data exist regarding the prevalence of respiratory viruses among healthcare associated pneumonia (HCAP) and hospital-acquired pneumonia (HAP) patients. Additionally, considering the narrow range of antiviral agents against respiratory viruses and the potential harm of invasive respiratory sampling for RT-PCR in critically ill patients, it is crucial to reveal in which patients should the sampling be performed, and whether such detection leads to a change in the clinical management or outcome of patients.

In this study, we aimed to identify the presence of common respiratory viral pathogens in patients with severe pneumonia who were admitted to the ICU, including those with CAP, HCAP, and HAP. In addition, we aimed to analyze the risk factors and clinical impact of such detection.

## Material and methods

### Study design and patients

We conducted a retrospective cohort study of adult patients who were admitted to a 22-bed medical ICU for severe pneumonia between January 1, 2008 and December 31, 2015. Pneumonia was diagnosed by the attending physician with the combination of a new lung infiltrate and clinical evidence including new onset fever, purulent sputum, leukocytosis, and a decline in oxygenation [10]. Pneumonia was categorized as CAP, HCAP, or HAP according to the American Thoracic Society and Infectious Disease Society of America guidelines [10, 11]. Respiratory specimen sampling for RT-PCR was performed when the attending physician considered it necessary for routine care.

This study was conducted in accordance with the amended Declaration of Helsinki. It was reviewed by the institutional review board of Seoul National University Hospital (protocol number: H-1603-106-750). This institutional review board approved this study. The requirement for informed consent was waived because all data were de-identified before analysis.

### Respiratory samples and multiplex PCR

RT-PCR analysis was performed when severe pneumonia did not respond to empirical antibacterial agents, when radiographic findings revealed ground glass opacities suggestive of atypical pathogens, or when patients were immune compromised. The decision regarding the type of specimen collected for RT-PCR analysis was made by the attending physician. Specimen collection included invasive (bronchoalveolar lavage [BAL]) and noninvasive (nasopharyngeal swab, sputum, or endotracheal aspirate) methods.

From 2008 to 2014, our institution utilized the Seeplex Respiratory Virus Detection assay (Seegene Inc., Seoul, Korea). This assay is based on the multiplex PCR method and uses a dual priming oligonucleotide system, which detects influenza virus types A and B, parainfluenza virus types 1, 2, and 3, RSV types A and B, adenovirus, metapneumovirus, coronavirus 229E, NL63 and OC43, and rhinovirus. After 2014, the Anyplex^TM^ II RV16 with Tagging Oligonucleotide Cleavage and Extension technology (Seegene, Seoul, Korea) was used. The RV16 uses 16 primer sets for the simultaneous detection of 16 respiratory viruses: influenza virus types A and B, parainfluenza virus types 1, 2, 3, and 4, RSV types A and B, adenovirus, metapneumovirus, coronavirus 229E, NL63 and OC43, rhinovirus, bocavirus, and enterovirus. Both assays are known to have good sensitivity and specificity [12–14]. However, due to South Korea’s national insurance policy, results for only 8 pathogens (influenza virus types A and B, parainfluenza virus types 1, 2, and 3, RSV types A and B, and adenovirus) were reported to the attending physicians throughout the study period.

### Variables and data collection

The baseline characteristics of patients, such as age, sex, Acute Physiology and Chronic Health Evaluation II (APACHE II) score, Charlson Comorbidity Index (CCI) score and underlying comorbidities, were reviewed. Patients with chronic obstructive pulmonary disease, asthma, bronchiectasis, pneumoconiosis, and tuberculosis destroyed lung were defined to have chronic lung disease [15–17]. The laboratory results analyzed included the white blood cell count, platelet count, C-reactive protein level, and clinical outcomes, including hospital length of stay (in days), ICU length of stay (in days), change of management, and in-hospital mortality. The causes of in-hospital mortality were also obtained from the medical records and death certificates of the patients.

Patients with and without virus detection were compared in terms of their baseline demographics, laboratory results, and clinical outcomes. Viral detection rates were described for each month and pneumonia category, and the pathogens of viral and bacterial coinfection were specified. Bacterial coinfection was considered to exist if both viruses and bacteria were detected, and bacteria were considered to be pathogens when blood cultures, respiratory tract specimen cultures, or urine pneumococcal antigens revealed positive results. Information about the impact on the clinical decision was obtained through a retrospective review of electronic medical records and orders from a communicating system.

### Statistical analysis

Categorical variables are reported as numbers and percentages, and continuous variables are reported as medians and interquartile ranges (IQR) or as means and standard deviations (SD), depending on their patterns of distribution. All statistical analyses were performed using IBM SPSS software (version 22.0; IBM Corp., Armonk, NY), and graphs were created using Prism 5 software (GraphPad software, San Diego, CA). A two-tailed *P*-value of less than 0.05 was considered statistically significant.

## Results

### Characteristics of patients with and without virus detection

During the 8-year study period, 2,347 patients were admitted to the medical ICU for severe pneumonia. Of these patients, 515 underwent RT-PCR for respiratory virus pathogens, 69 (13.4%) of whom had positive results.

**Fig 1:**
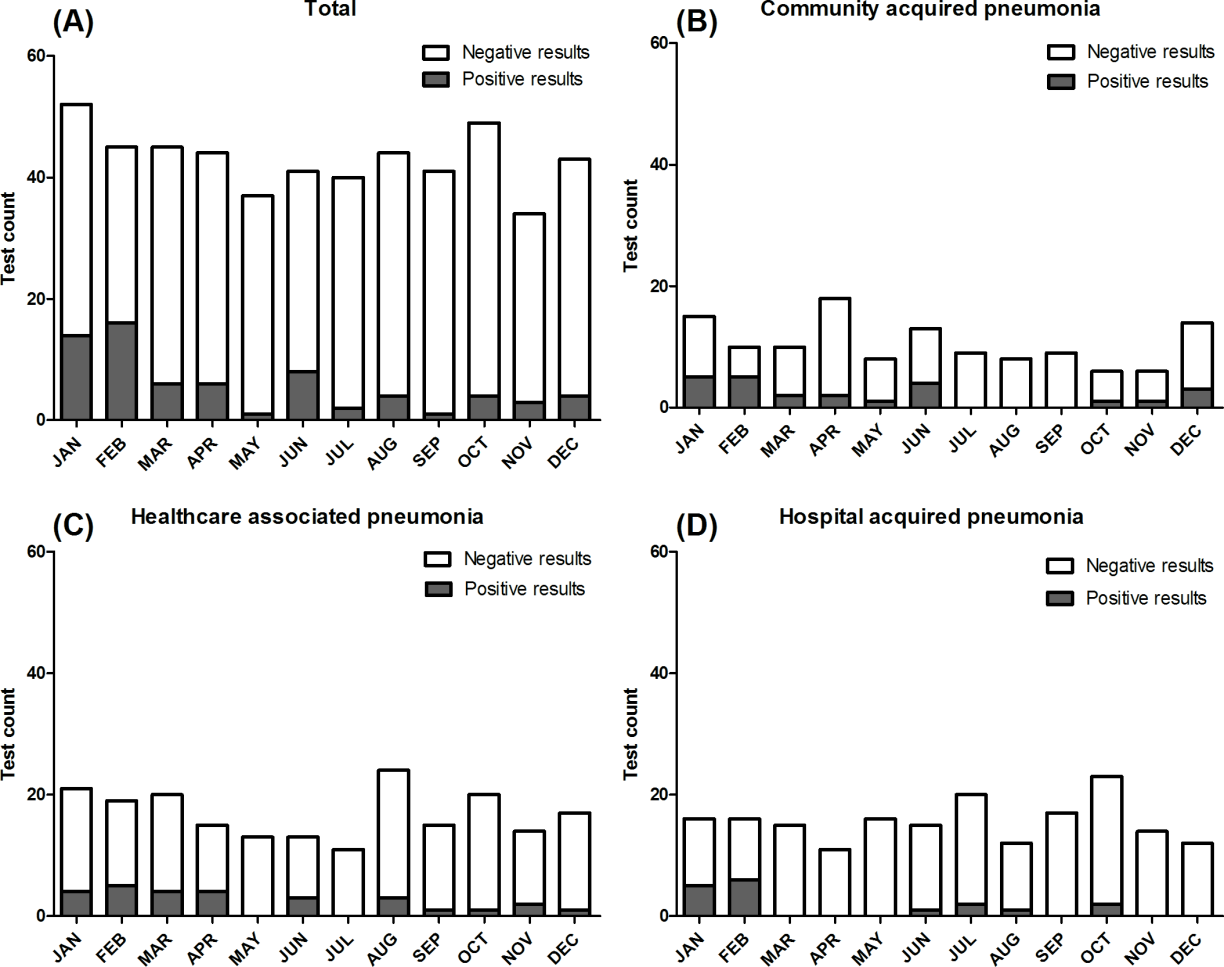
Detection of respiratory viruses by month and pneumonia category. The shaded bars represent positive results, and the white bars represent negative results. Detection rate was highest in February and January. (A) Total patients. (B) Community acquired pneumonia patients. (C) Healthcare associated pneumonia patients. (D) Hospital acquired pneumonia patients.

The patients with and without respiratory virus detection showed no significant differences in terms of age, sex, and disease severity, which was presented as the APACHE II score. However, patients with a community onset, history of recent (<2 weeks) chemotherapy, and lower platelet count (<200,000/μL) had higher rates of virus detection. Additionally, the rate of RT-PCR detection of respiratory viral pathogens showed seasonality: the rate was higher during February (35.6%) and January (26.7%) and was lower in September (2.5%) and May (2.7%). Seasonality was more prominent with CAP, compared to HCAP and HAP (Fig 1). Clinical outcomes, such as the length of hospital stay, length of ICU stay, and in-hospital mortality, did not differ between the two groups (Table 1).

**Table 1:** Demographics and clinical outcomes of patients undergone reverse transcriptase polymerase chain reaction for respiratory viruses.

Variables	Virus detected n = 69	Virus not detected n = 446	*P*
Age	64 (56–73)	65 (56–73)	0.934
Sex, male	57 (82.6)	393 (88.1)	0.200
APACHE II score	27.23±1.15	27.34±0.36	0.918
Pneumonia category			0.039
Community acquired pneumonia	24 (34.8)	102 (22.9)	
Healthcare associated pneumonia	28 (40.6)	174 (39.0)	
Hospital acquired pneumonia	17 (24.6)	170 (38.1)	
Charlson comorbidity index with age	4 (3–6)	4 (3–6)	0.445
Comorbidities			
Solid organ malignancy	15 (21.7)	135 (30.3)	0.147
Diabetes mellitus	13 (18.8)	96 (21.5)	0.611
Hematologic malignancy	19 (27.5)	79 (17.7)	0.053
Use of immune suppressants	15 (21.7)	73 (16.4)	0.270
Chronic kidney disease	13 (18.8)	61 (13.7)	0.255
Chronic lung disease*	6 (8.7)	48 (10.8)	0.602
Recent chemotherapy	15 (21.7)	42 (9.4)	0.002
Specimen for RT-PCR			<0.001
Invasive method only	36 (52.2)	352 (78.9)	
Noninvasive method only	8 (11.6)	37 (8.3)	
Both	25 (36.2)	57 (12.8)	
Laboratory findings			
Low white blood cell count (<4,000/μL)	15 (21.7)	69 (15.5)	0.259
Low platelet count (<200,000/μL)	57 (82.6)	291 (65.2)	0.006
C-reactive protein (mg/dL)	15.3 (7.7–21.6)	13.5 (6.9–22.3)	0.379
Bronchoalveolar lavage fluid			
Segmented neutrophils	58 (16–79)	58 (22–78)	0.935
Lymphocytes	10 (3–26)	11 (5–24)	0.726
CD4/CD8 ratio	0.80 (0.32–1.27)	0.91 (0.54–1.78)	0.163
Length of hospital stay	31 (22–46)	33 (19–58)	0.602
Length of intensive care unit stay	12 (7–20)	11 (6–19)	0.899
In-hospital mortality			
Any	42 (60.9)	281 (63.0)	0.733
Pneumonia associated	28 (66.7)	112 (63.3)	0.681

*Chronic lung disease defined as chronic obstructive pulmonary disease, asthma, bronchiectasis, pneumoconiosis, and tuberculosis destroyed lung.

Values are presented as number (percentage), median (interquartile range), or mean ± standard deviation. APACHE II, Acute Physiology and Chronic Health Evaluation II; RT-PCR, reverse transcription polymerase chain reaction

The detection rate was the highest when both invasive and noninvasive samplings were performed (25 among 82 patients, 30.5%). Among the 25 patients who underwent both types of sampling, new information was acquired from additional invasive sampling in 7 patients. Additional information was not obtained in the other 18 patients: 10 patients had the same results from invasive and noninvasive sampling, and 8 patients had negative results from the additional invasive sampling.

### Pathogens for severe pneumonia

Among the 69 patients with positive RT-PCR results, RSV A was the most common pathogen detected (n = 21), followed by influenza A (n = 18), parainfluenza 3 (n = 12), RSV B (n = 9), adenovirus (n = 7), influenza B (n = 3), and parainfluenza 1 (n = 2). The detection rates showed similar distributions when they were divided into the 3 pneumonia categories, except for that of influenza A, which tended to be more prevalent in community settings (Table 2).

**Table 2:** Types of respiratory virus detected according to each pneumonia categories.

Type of respiratory virus	Total n = 515	CAP n = 126	HCAP n = 202	HAP n = 187	*P*
Respiratory syncytial virus A	21 (4.1)	8 (6.4)	9 (4.5)	4 (2.1)	0.171
Influenza A	18 (3.5)	8 (6.4)	7 (3.5)	3 (1.6)	0.081
Parainfluenza 3	12 (2.3)	3 (2.4)	5 (2.5)	4 (2.1)	>0.999
Respiratory syncytial virus B	9 (1.8)	3 (2.4)	2 (1.0)	4 (2.1)	0.528
Adenovirus	7 (1.4)	1 (0.8)	3 (1.5)	3 (1.6)	0.899
Influenza B	3 (0.6)	2 (1.6)	1 (0.5)	0 (0.0)	0.257
Parainfluenza 1	2 (0.4)	0 (0.0)	2 (1.0)	0 (0.0)	0.344

Values are presented as number (percentage). CAP, community acquired pneumonia; HCAP, healthcare associated pneumonia; HAP, hospital acquired pneumonia

Bacterial coinfection was detected in 27 (39.1%) patients. The most common bacterial pathogens were *Staphylococcus aureus* (n = 8), followed by *Enterococcus faecium* (n = 4), *Klebsiella pneumoniae* (n = 4), *Acinetobacter baumannii* (n = 2), and *Pseudomonas aeruginosa* (n = 2). Bacteria were most commonly detected when influenza A was confirmed (n = 11), followed by RSV A (n = 7), parainfluenza 3 (n = 4), adenovirus (n = 3), RSV B (n = 2), and influenza B (n = 1) (Table 3). Patients with bacterial coinfection had a higher neutrophil count, lower lymphocyte count, and lower CD4/CD8 ratio from the BAL fluid than those without coinfection. Otherwise, the two groups did not show significant differences in their demographics and clinical outcomes (S1 Table).

**Table 3:** Pathogens of viral and bacterial coinfection.

Bacteria of coinfection	Influenza A	RSV A	Parainfluenza 3	Adenovirus	RSV B	Influenza B	Total
Gram positive	7	5	1	1	1	1	15
*Staphylococcus aureus*	5	-	1	1	-	1	8
*Enterococcus faecium*	1	3	-	-	-	-	4
*Staphylococcus epidermidis*	1	1	-	-	-	-	1
*Streptococcus viridans*	-	1	-	-	-	-	1
*Corynebacterium striatum*	-	-	-	-	1	-	1
Gram negative	4	2	3	2	1	-	12
*Klebsiella pneumoniae*	1	1	-	-	1	-	4
*Acinetobacter baumannii*	1	-	-	1	-	-	2
*Pseudomonas aeruginosa*	-	1	1	-	-	-	2
*Klebsiella oxytoca*	1	-	-	-	-	-	1
*Escherichia coli*	1	-	-	-	-	-	1
*Moraxella catarrhalis*	-	-	1	-	-	-	1
*Stenotrophomonas maltophilia*	-	-	1	1	-	-	1
Total	11	7	4	3	2	1	

Values are presented as numbers.

### Clinical impact of virus detection

The detection of a viral pathogen led to changes in the management of the disease in 23 (33.3%) patients. Twelve patients received antiviral therapy such as oseltamivir and ribavirin, and empirical antiviral therapy was continued or extended in 4 patients. The use of immunosuppressive agents, including steroids, was decreased or stopped in 3 patients. In some patients, antibiotics (n = 2) or antiviral agents (n = 1) were discontinued because bacterial pathogen was no longer suspected, or the detected virus had no effective antiviral agent (Table 4).

**Table 4:** Impact of respiratory virus detection on clinical decision among the patients in intensive care unit.

Variables	Values
Change of clinical management	23 (33.3)
Addition of antiviral therapy	12 (17.4)
Continue or extend empirical antiviral therapy	4 (5.8)
Reduction or cessation of immunosuppressant	3 (4.3)
Stop antibiotics	2 (2.9)
Change antiviral agent	1 (1.4)
Stop antiviral agent	1 (1.4)
No change of clinical management	46 (66.7)

Values are presented as number (percentage).

The clinical outcomes, such as the length of hospital stay, length of ICU stay, and in-hospital mortality, were compared according to changes in management. However, the differences were not statistically significant (S2 Table).

## Discussion

Our study identified the risk factors, prevalence, and clinical impact of virus detection among patients with severe pneumonia who were admitted to the medical ICU. Respiratory viral infection should be suspected in patients from the community, during the winter season, in patients with recent chemotherapy, and in patients with a low serum platelet count. The overall virus detection rate was 13.3%, with RSV A being the most common pathogen and influenza A virus being the most common pathogen of bacterial coinfection. Such detection of respiratory viruses led to changes in management in one-third of the patients.

The virus detection rate was higher in February and January as well as in patients with a community onset. This finding supports the recommendation to use empirical therapy against influenza virus during the winter season for hospitalized CAP patients [18]. Patients with recent chemotherapy were at a higher risk for viral pneumonia, which is consistent with previous knowledge that immunosuppression is a risk factor for influenza viral pneumonia [19]. The virus detection rate was highest in patients who had undergone both invasive and noninvasive sampling (n = 25), and more information was obtained from further invasive sampling in approximately 28% of the patients (n = 7). Although the potential harm of BAL in critically ill patients must be thoroughly reviewed before the procedure, further invasive samplings should be considered in selected patients stated above (winter seasons, community onset, recent chemotherapy, low platelet count, and so on) for additional virus detection.

RSV, an important pathogen that can result in severe pneumonia, especially in the elderly, was the most common pathogen detected [20]. Previous studies have differed in the detailed distribution of pathogens, but many have reported that the most common viral pathogens include influenza, parainfluenza, and RSV [21–23]. Considering the limited strategies for treating and preventing respiratory viruses other than influenza, this distribution of various pathogens may further emphasize the need for the development of novel antiviral agents and vaccines.

This study is the first to specify the clinical impact of adult-onset severe viral pneumonia according to the detection of respiratory viruses. Previous studies have been conducted in children or with milder forms of pneumonia [24]. However, children have much higher rates of respiratory viral illnesses than adults and should be discussed separately, and severe pneumonia is of most interest in the ICU setting [6, 25]. Among the 23 patients whose management was changed, the most common change was in antiviral agents (n = 18). Currently, anti-influenza agents are the only actively used antiviral agents, and ribavirin is the only antiviral treatment option for non-influenza respiratory viruses [26]. Our study results emphasize the need for the development of novel antiviral agents against respiratory viruses. Apart from antiviral agents, respiratory viral detection in critically ill patients led to a reduction or cessation of immunosuppressant treatment in 3 patients. The use of high-dose steroids is known to be associated with a higher mortality rate and longer viral shedding in influenza A patients [27]. Therefore, it can be helpful to reduce the use of immune-modulating agents, including steroids, to improve patient outcomes. Two other patients stopped using empirical antibiotics, and their treatment focused on the respiratory viruses as pathogens. The long-term use of antibacterial agents in patients with viral pneumonia is known to increase the risk for developing multidrug-resistant pathogens and *Clostridium difficile* infection rather than improving clinical outcomes [28, 29].

The bacterial coinfection rate of the present study was similar to that of previous reports [8, 30], which further supports the fact that patients with viral infection should be carefully examined for any additional bacterial infection. The most common bacterial pathogens of coinfection were common colonizers of the nasopharynx [31]. However, we failed to show a significant difference in mortality related to coinfection. This result is also consistent with previous reports, which showed comparable results for patients with and without bacterial coinfection [32]. The consequences of bacterial coinfection require further study.

Our study has several limitations. First, it was a retrospective study performed in a single center. Second, we considered all detected microorganisms as pathogens. However, a detected respiratory virus was unlikely to be neutral and was pathogenic in certain group of patients according to a previous report [33]. Although further studies are required, the possibility of invasiveness of detected respiratory virus should be taken into account. Third, although the RT-PCR kit of our institution is known to have good sensitivity and specificity [12–14], the risk of false-negativity cannot be perfectly ruled out. Fourth, the detection rate was lower than that of previous reports [21, 22]. The limited number of reported pathogens and inclusion of HAP may be responsible for this result. Of the 519 patients who underwent multiplex RT-PCR detection of respiratory viral pathogens, 188 (36.2%) were HAP patients. The detection rate increased to 18.8% when only CAP patients were considered, which is comparable to the results from a recent systematic review [34].

In conclusion, non-influenza respiratory viruses were commonly detected in severe pneumonia patients, and the detection of viral pathogens in patients with severe pneumonia can lead to changes in clinical management strategies. Therefore, RT-PCR analysis should be actively performed for severe pneumonia in the ICU, especially among those with risk factors for viral infection. Furthermore, future efforts are required to develop novel antiviral agents for non-influenza respiratory viruses.

## Supporting information

S1 Table Characteristics and clinical outcomes of viral pneumonia patients with and without bacterial coinfection. (DOCX)

S2 Table Clinical outcomes according to change in clinical management after detection of respiratory viruses. (DOCX)

S1 File Anonymized minimal dataset of the study. (XLSX)

## Abbreviations

RSV: respiratory syncytial virus
RT-PCR: reverse transcription-polymerase chain reaction
ICU: intensive care unit
CAP: community acquired pneumonia
HCAP: healthcare associated pneumonia
HAP: hospital-acquired pneumonia
BAL: bronchoalveolar lavage
APACHE II: Acute Physiology and Chronic Health Evaluation II
CCI: Charlson Comorbidity Index
IQR: interquartile range
SD: standard deviation

## References

[1] Jain S, Self WH, Wunderink RG, Fakhran S, Balk R, Bramley AM, et al Community-Acquired Pneumonia Requiring Hospitalization among U.S. Adults. N Engl J Med. 2015;373(5):415–427. doi: 10.1056/NEJMoa1500245 26172429 10.1056/NEJMoa1500245 PMC4728150

[2] Falsey AR, McElhaney JE, Beran J, van Essen GA, Duval X, Esen M, et al Respiratory syncytial virus and other respiratory viral infections in older adults with moderate to severe influenza-like illness. J Infect Dis. 2014;209(12):1873–1881. doi: 10.1093/infdis/jit839 24482398 10.1093/infdis/jit839 PMC4038137

[3] Wiemken T, Peyrani P, Bryant K, Kelley RR, Summersgill J, Arnold F, et al Incidence of respiratory viruses in patients with community-acquired pneumonia admitted to the intensive care unit: results from the Severe Influenza Pneumonia Surveillance (SIPS) project. Eur J Clin Microbiol Infect Dis. 2013;32(5):705–710. doi: 10.1007/s10096-012-1802-8 23274861 10.1007/s10096-012-1802-8

[4] Cunha BA, Corbett M, Mickail N. Human parainfluenza virus type 3 (HPIV 3) viral community-acquired pneumonia (CAP) mimicking swine influenza (H1N1) during the swine flu pandemic. Heart Lung. 2011;40(1):76–80. doi: 10.1016/j.hrtlng.2010.05.060 20888645 10.1016/j.hrtlng.2010.05.060

[5] Aramburo A, van Schaik S, Louie J, Boston E, Messenger S, Wright C, et al Role of real-time reverse transcription polymerase chain reaction for detection of respiratory viruses in critically ill children with respiratory disease: Is it time for a change in algorithm? Pediatr Crit Care Med. 2011;12(4):e160–165. doi: 10.1097/PCC.0b013e3181f36e86 20711084 10.1097/PCC.0b013e3181f36e86

[6] Subramony A, Zachariah P, Krones A, Whittier S, Saiman L. Impact of Multiplex Polymerase Chain Reaction Testing for Respiratory Pathogens on Healthcare Resource Utilization for Pediatric Inpatients. J Pediatr. 2016;173:196–201 e192. doi: 10.1016/j.jpeds.2016.02.050 27039227 10.1016/j.jpeds.2016.02.050 PMC5452417

[7] Nguyen C, Kaku S, Tutera D, Kuschner WG, Barr J. Viral Respiratory Infections of Adults in the Intensive Care Unit. J Intensive Care Med. 2016;31(7):427–441. doi: 10.1177/0885066615585944 25990273 10.1177/0885066615585944

[8] Choi SH, Hong SB, Ko GB, Lee Y, Park HJ, Park SY, et al Viral infection in patients with severe pneumonia requiring intensive care unit admission. Am J Respir Crit Care Med. 2012;186(4):325–332. doi: 10.1164/rccm.201112-2240OC 22700859 10.1164/rccm.201112-2240OC

[9] Micek ST, Chew B, Hampton N, Kollef MH. A Case-Control Study Assessing the Impact of Nonventilated Hospital-Acquired Pneumonia on Patient Outcomes. Chest. 2016;150(5):1008–1014. doi: 10.1016/j.chest.2016.04.009 27102181 10.1016/j.chest.2016.04.009 PMC7094544

[10] Kalil AC, Metersky ML, Klompas M, Muscedere J, Sweeney DA, Palmer LB, et al Management of Adults With Hospital-acquired and Ventilator-associated Pneumonia: 2016 Clinical Practice Guidelines by the Infectious Diseases Society of America and the American Thoracic Society. Clin Infect Dis. 2016. 10.1093/cid/ciw353 PMC4981759 27418577

[11] American Thoracic S, Infectious Diseases Society of A. Guidelines for the management of adults with hospital-acquired, ventilator-associated, and healthcare-associated pneumonia. Am J Respir Crit Care Med. 2005;171(4):388–416. doi: 10.1164/rccm.200405-644ST 15699079 10.1164/rccm.200405-644ST

[12] Kim SR, Ki CS, Lee NY. Rapid detection and identification of 12 respiratory viruses using a dual priming oligonucleotide system-based multiplex PCR assay. J Virol Methods. 2009;156(1–2):111–116. doi: 10.1016/j.jviromet.2008.11.007 19063921 10.1016/j.jviromet.2008.11.007 PMC7112863

[13] Drews SJ, Blair J, Lombos E, DeLima C, Burton L, Mazzulli T, et al Use of the Seeplex RV Detection kit for surveillance of respiratory viral outbreaks in Toronto, Ontario, Canada. Ann Clin Lab Sci. 2008;38(4):376–379. 18988931

[14] Cho CH, Chulten B, Lee CK, Nam MH, Yoon SY, Lim CS, et al Evaluation of a novel real-time RT-PCR using TOCE technology compared with culture and Seeplex RV15 for simultaneous detection of respiratory viruses. J Clin Virol. 2013;57(4):338–342. doi: 10.1016/j.jcv.2013.04.014 23743345 10.1016/j.jcv.2013.04.014 PMC7108272

[15] Viglio S, Iadarola P, Lupi A, Trisolini R, Tinelli C, Balbi B, et al MEKC of desmosine and isodesmosine in urine of chronic destructive lung disease patients. Eur Respir J. 2000;15(6):1039–1045. 10885422 10.1034/j.1399-3003.2000.01511.x

[16] Crestanello JA, Higgins RS, He X, Saha-Chaudhuri P, Englum BR, Brennan JM, et al The association of chronic lung disease with early mortality and respiratory adverse events after aortic valve replacement. Ann Thorac Surg. 2014;98(6):2068–2077. doi: 10.1016/j.athoracsur.2014.06.087 25443011 10.1016/j.athoracsur.2014.06.087

[17] Oliveira EC, Marik PE, Colice G. Influenza pneumonia: a descriptive study. Chest. 2001;119(6):1717–1723. 11399696 10.1378/chest.119.6.1717

[18] McCracken JP, Prill MM, Arvelo W, Lindblade KA, Lopez MR, Estevez A, et al Respiratory syncytial virus infection in Guatemala, 2007–2012. J Infect Dis. 2013;208 Suppl 3:S197–206. 24265479 10.1093/infdis/jit517

[19] Rello J, Pop-Vicas A. Clinical review: primary influenza viral pneumonia. Crit Care. 2009;13(6):235 doi: 10.1186/cc8183 20085663 10.1186/cc8183 PMC2811908

[20] Falsey AR, Hennessey PA, Formica MA, Cox C, Walsh EE. Respiratory syncytial virus infection in elderly and high-risk adults. N Engl J Med. 2005;352(17):1749–1759. doi: 10.1056/NEJMoa043951 15858184 10.1056/NEJMoa043951

[21] Tramuto F, Maida CM, Napoli G, Mammina C, Casuccio A, Cala C, et al Burden and viral aetiology of influenza-like illness and acute respiratory infection in intensive care units. Microbes Infect. 2016;18(4):270–276. doi: 10.1016/j.micinf.2015.11.008 26706819 10.1016/j.micinf.2015.11.008 PMC7129629

[22] Das D, Le Floch H, Houhou N, Epelboin L, Hausfater P, Khalil A, et al Viruses detected by systematic multiplex polymerase chain reaction in adults with suspected community-acquired pneumonia attending emergency departments in France. Clin Microbiol Infect. 2015;21(6):608 e601–608. 25704448 10.1016/j.cmi.2015.02.014 PMC7128919

[23] Hong HL, Hong SB, Ko GB, Huh JW, Sung H, Do KH, et al Viral infection is not uncommon in adult patients with severe hospital-acquired pneumonia. PLoS One. 2014;9(4):e95865 doi: 10.1371/journal.pone.0095865 24752070 10.1371/journal.pone.0095865 PMC3994115

[24] Mayer LM, Kahlert C, Rassouli F, Vernazza P, Albrich WC. Impact of viral multiplex real-time PCR on management of respiratory tract infection: a retrospective cohort study. Pneumonia (Nathan). 2017;9:4. 28702306 10.1186/s41479-017-0028-z PMC5471894

[25] Rogers BB, Shankar P, Jerris RC, Kotzbauer D, Anderson EJ, Watson JR, et al Impact of a rapid respiratory panel test on patient outcomes. Arch Pathol Lab Med. 2015;139(5):636–641. doi: 10.5858/arpa.2014-0257-OA 25152311 10.5858/arpa.2014-0257-OA

[26] Waghmare A, Englund JA, Boeckh M. How I treat respiratory viral infections in the setting of intensive chemotherapy or hematopoietic cell transplantation. Blood. 2016;127(22):2682–2692. doi: 10.1182/blood-2016-01-634873 26968533 10.1182/blood-2016-01-634873 PMC4891952

[27] Cao B, Gao H, Zhou B, Deng X, Hu C, Deng C, et al Adjuvant Corticosteroid Treatment in Adults With Influenza A (H7N9) Viral Pneumonia. Crit Care Med. 2016;44(6):e318–328. doi: 10.1097/CCM.0000000000001616 26934144 10.1097/CCM.0000000000001616

[28] Crotty MP, Meyers S, Hampton N, Bledsoe S, Ritchie DJ, Buller RS, et al Impact of antibacterials on subsequent resistance and clinical outcomes in adult patients with viral pneumonia: an opportunity for stewardship. Crit Care. 2015;19:404 doi: 10.1186/s13054-015-1120-5 26577540 10.1186/s13054-015-1120-5 PMC4650137

[29] Shorr AF, Zilberberg MD. Going Viral: Importance of Viral Pathogens in Nonventilated Hospital-Acquired Pneumonia. Chest. 2016;150(5):991–992. doi: 10.1016/j.chest.2016.05.028 27832891 10.1016/j.chest.2016.05.028

[30] Falsey AR, Becker KL, Swinburne AJ, Nylen ES, Formica MA, Hennessey PA, et al Bacterial complications of respiratory tract viral illness: a comprehensive evaluation. J Infect Dis. 2013;208(3):432–441. doi: 10.1093/infdis/jit190 23661797 10.1093/infdis/jit190 PMC3699009

[31] Chertow DS, Memoli MJ. Bacterial coinfection in influenza: a grand rounds review. Jama. 2013;309(3):275–282. doi: 10.1001/jama.2012.194139 23321766 10.1001/jama.2012.194139

[32] Karhu J, Ala-Kokko TI, Vuorinen T, Ohtonen P, Syrjala H. Lower respiratory tract virus findings in mechanically ventilated patients with severe community-acquired pneumonia. Clin Infect Dis. 2014;59(1):62–70. doi: 10.1093/cid/ciu237 24729498 10.1093/cid/ciu237 PMC4305142

[33] Zhan Y, Yang Z, Chen R, Wang Y, Guan W, Zhao S. Respiratory virus is a real pathogen in immunocompetent community-acquired pneumonia: comparing to influenza like illness and volunteer controls. BMC Pulm Med. 2014;14:144 doi: 10.1186/1471-2466-14-144 25178477 10.1186/1471-2466-14-144 PMC4236731

[34] Alimi Y, Lim WS, Lansbury L, Leonardi-Bee J, Nguyen-Van-Tam JS. Systematic review of respiratory viral pathogens identified in adults with community-acquired pneumonia in Europe. J Clin Virol. 2017;95:26–35. doi: 10.1016/j.jcv.2017.07.019 28837859 10.1016/j.jcv.2017.07.019 PMC7185624

