# Supplementary material for: Respiratory virus of severe pneumonia in South Korea: Prevalence and clinical implications
Source: PLoS One. 2018 Jun 18;13(6):e0198902. doi: 10.1371/journal.pone.0198902 (PMC6005478; doi:10.1371/journal.pone.0198902)
Supplement: S1 Table — (DOCX) [file pone.0198902.s001.docx]

**S1 Table.** Characteristics and clinical outcomes of viral pneumonia patients with and without bacterial coinfection

| Variables | With Bacterial coinfection  n = 27 | Without Bacterial coinfection  n = 42 | *P* |
| --- | --- | --- | --- |
| Age | 65 (56–73) | 63 (56–74) | 0.539 |
| Gender, male | 20 (74.1) | 37 (88.1) | 0.134 |
| Pneumonia category |  |  | 0.918 |
| Community acquired pneumonia | 10 (37.0) | 14 (33.3) |  |
| Healthcare-associated pneumonia | 11 (40.7) | 17 (40.5) |  |
| Hospital acquired pneumonia | 6 (22.2) | 11 (26.2) |  |
| APACHE II score | 30 (24–35) | 27 (20–33) | 0.184 |
| Charlson comorbidity score with age | 4 (3–6) | 4 (2–6) | 0.441 |
| Comorbidities |  |  |  |
| Solid organ malignancy | 6 (22.2) | 9 (21.4) | >0.999 |
| Diabetes mellitus | 5 (18.5) | 8 (19.1) | >0.999 |
| Hematologic malignancy | 8 (29.6) | 11 (26.2) | 0.755 |
| Use of immune suppressants | 4 (18.1) | 11 (26.2) | 0.373 |
| Chronic kidney disease | 6 (22.2) | 7 (16.7) | 0.565 |
| Chronic lung disease | 4 (14.8) | 2 (4.76) | 0.201 |
| Recent chemotherapy | 6 (22.2) | 9 (21.4) | >0.999 |
| Laboratory data |  |  |  |
| White blood cell count (x10^3^/μL) | 10.01 (6.81–15.0) | 10.68 (5.45–16.09) | 0.754 |
| Platelet count (x10^3^/μL) | 66 (34–179) | 128 (57–188) | 0.212 |
| C-reactive protein (mg/dL) | 15.5 (7.9–23.6) | 14.9 (7.7–20.6) | 0.638 |
| Bronchoalveolar lavage fluid |  |  |  |
| Segmented neutrophils | 80 (59–90) | 40 (9–63) | 0.006 |
| Lymphocytes | 7 (3–12) | 18 (3–37) | 0.014 |
| CD4/CD8 ratio | 0.80 (0.32–1.27) | 0.95 (0.71–1.38) | 0.040 |
| Length of hospital stay | 30 (19–50) | 32 (23–46) | 0.685 |
| Length of intensive care unit stay | 14 (6–23) | 11 (7–17) | 0.763 |
| In-hospital mortality |  |  |  |
| Any | 17 (63.0) | 25 (59.5) | 0.775 |
| Pneumonia associated | 12 (44.4) | 16 (38.1) | 0.600 |

Values are presented as number (percentage), median (interquartile range), or mean ± standard deviation. APACHE II, Acute Physiology and Chronic Health Evaluation II.
