# Supplementary material for: Respiratory virus of severe pneumonia in South Korea: Prevalence and clinical implications
Source: PLoS One. 2018 Jun 18;13(6):e0198902. doi: 10.1371/journal.pone.0198902 (PMC6005478; doi:10.1371/journal.pone.0198902)
Supplement: S2 Table — (DOCX) [file pone.0198902.s002.docx]

**S2 Table.** Clinical outcomes according to change in clinical management after detection of respiratory viruses

| **Variables** | **Patients without impact on**  **clinical decision n = 46** | **Patients with impact on clinical decision n = 23** | ***P*** |
| --- | --- | --- | --- |
| Length of hospital stay | 32 (20–49) | 29 (22–46) | 0.990 |
| Length of intensive care unit stay | 9 (6–18) | 15 (8–22) | 0.070 |
| In-hospital mortality |  |  |  |
| Any | 26 (56.5) | 16 (69.6) | 0.295 |
| Pneumonia associated | 16 (34.8) | 12 (52.2) | 0.165 |

Values are presented as number (percentage) or median (interquartile range).
